# Clinical Epidemiology of Mineral Bone Disorder Markers in Prevalent Hemodialysis Patients in the Xinjiang Uyghur Autonomous Region in China

**DOI:** 10.1155/2017/2516934

**Published:** 2017-02-19

**Authors:** Ying-Ping Sun, Wen-Jun Yang, Su-Hua Li, Yuan-yuan Han, Jian Liu

**Affiliations:** Department of Nephrology, The First Affiliated Hospital of Xinjiang Medical University, Xinjiang, China

## Abstract

We investigated the clinical epidemiology of mineral bone disorder markers in prevalent hemodialysis (HD) patients in Xinjiang, the largest province in China. Data were obtained from 59 hospitals. A total of 3725 patients tracked from January 1 to December 31, 2014, were enrolled. Serum calcium (Ca) levels, phosphorus (P) levels, and intact parathyroid hormone (iPTH) levels were analyzed. Serum Ca levels were lower compared to the International Dialysis Outcomes and Practice Patterns Study (DOPPS4) and the Chinese DOPPS. The hypercalcemia rate was similar to DOPPS4 and lower than in the Chinese DOPPS. Serum P levels were higher than in DOPPS4 and lower than those in the Chinese DOPPS. Hyperphosphatemia rates were higher than DOPPS4 and lower than Chinese DOPPS. Serum iPTH levels were higher than in DOPPS4 and the Chinese DOPPS. We demonstrated higher serum P and iPTH levels in Xinjiang HD patients than in the DOPPS4 and Chinese DOPPS. In contrast, serum Ca levels were lower than the other two studies. High hypocalcemia and hyperphosphatemia rates may suggest that HD services in Xinjiang are inadequate. A multidiscipline chronic kidney disease (CKD) care program needs to be established to improve chronic kidney disease-mineral and bone disorder (CKD-MBD) target achievement in Xinjiang.

## 1. Introduction

Xinjiang is the largest province (1.64 million square kilometers) in China, located in the northwest with a total population of about 23 million (2015). Xinjiang is further away from the ocean than any other place on earth and is inhabited by Uyghur and Han people, and 11 other ethnic groups. The characteristics of the geographical environment and lifestyle are significantly different to that of tropical and subtropical residents.

The prevalence of chronic kidney disease in rural areas was 5.4% (2007) and 9.6% (2010) in urban areas [1, 2], and an estimated 1-2 million patients were suffering from end-stage renal disease (ESRD). In 2010, the Chinese Society of Nephrology established the nationwide renal data registration platform. This platform, known as the Chinese National Renal Data System (CNRDS), collects demographic, clinical, and laboratory data on dialysis patients [3]. The Xinjiang Quality Control Center for dialysis patients is one of the registered centers in the CNRDS. Owing to the specific habitual styles of different ethnic groups and the geographical size of Xinjiang, developing a strategy for chronic kidney disease management is challenging for nephrologists. In the present study, we report on the status of chronic kidney disease-mineral bone disorder (CKD-MBD) in Xinjiang based on the registered data from the CNRDS.

## 2. Materials and Methods

### 2.1. Database

Registered data from the Xinjiang Quality Control of Dialysis Patients database covers nearly all inpatient and outpatient dialysis medical records in the Xinjiang area. Data for the present study were obtained from 14 tertiary-level hospitals and 45 secondary-level hospitals in Xinjiang. Patients were tracked from January 1 to December 31, 2014. Enrollment criteria included (1) use of dialysis, (2) being over the age of 18, (3) use of hemodialysis (HD) for at least three months, and (4) available baseline serum calcium (Ca), phosphorus (P), and intact parathyroid hormone (iPTH) data obtained between January 1 and December 31, 2014. Exclusion criteria were (1) comorbidities with malignancy, active infectious diseases, severe liver diseases, or liver cirrhosis and (2) acute kidney failure or chronic kidney failure with temporary dialysis. All participants received more than one fasting blood sampling for laboratory examinations in the study period. A mean value of each laboratory parameter in individual was used in statistical analysis. We confirmed hypocalcemia as a total serum calcium level of < 8.4 mg/dL, hypercalcemia as a total serum calcium level of > 9.5 mg, hyperphosphatemia as a serum phosphorus level of > 5.5 mg/L, and high iPTH with a serum iPTH level of > 300 pg/mL. The management of CKD-MBD was based on the Kidney Disease: Improving Global Outcomes (KDIGO) guidelines implemented in 2009 [4]. The data collected for Xinjiang were compared to data from the CNRDS, DOPPS4, and the Chinese DOPPS [5, 6].

All blood samples were evaluated using commercial kits and an autoanalyzer. Albumin levels were measured using the bromocresol green (BCG) method (Beckman Coulter, Inc., USA). Serum phosphorus and serum calcium were measured by spectrophotometry assay (Beckman Coulter, Inc., USA; Roche Inc., Swiss). iPTH level was measured by electrochemiluminescence immunoassay (Roche Inc., Swiss). The urea reduction ratio (URR) was calculated using the following equation: URR = [predialysis blood urea nitrogen (BUN) – postdialysis BUN/predialysis BUN] × 100%. The urea clearance index (*Kt*/*V*) was calculated using the following equation: *Kt*/*V* urea = −Ln(*R* − 0.008 × *t*)+[4 − (3.5 × *R*)] × UF/*W*, where *R* is the ratio of postdialysis and predialysis serum urea nitrogen content, *t* is the duration of dialysis (h), ultrafiltration (UF) is the ultrafiltrate amount (L), and *W* is the postdialysis body weight (kg).

This study was conducted in accordance with the Declaration of Helsinki (1964). The requirement for obtaining informed consent from study patients was waived in accordance with the retrospective data review regulations of the Committee on Human Research at Hospital.

### 2.2. Statistical Analysis

SPSS 17.0 statistical software was used for data analysis. Continuous data are presented as mean ± standard deviation (SD), and categorical variables are expressed as frequency counts and percentages. Box plot was used for visual presentation of continuous variables and the median 75th and 25th percentiles. The Mann–Whitney *U* test was used for comparisons between groups. The chi-square test and one-way ANOVA were used to analyze the associations between categorical and continuous variables, respectively. Statistical significance was set at *P* < 0.05.

## 3. Results

A total of 59 hospital-facilitated HD units participated in this study. This covered 80% of the whole population of Xinjiang in 2014. Data on 3725 patients (Hans 2352 (63.2%) and Uyghurs 1373 (36.8%)) were collected. The prevalence of end-stage renal diseases (ESRD) was 162 people per million people (pmp) in the total population, 235 pmp for the Han group, and 137 pmp for the Uyghur group.  Table 1 shows the general demographic characteristics of the study cohort. The mean patient age was 52.09 years, and 64.9% of participants were male. Considering the study cohort as a whole, the main causes of ESRD were primary glomerulonephritis (42.4%), diabetic nephropathy (23.1%), and hypertension-related kidney diseases (15.4%). In comparison, causes of ESRD in the Han and Uyghur groups were glomerulonephritis (37.0% versus 51.8%) and diabetic nephropathy (25.7% versus 18.6%). The proportion of dialysis vintages of < 1 year, 1–3 years, 3–5 years, 5–10 years, and > 10 years were 24.5%, 41.4%, and 20.3%, 12.0%, and 1.8%, respectively.

**Figure 1:**
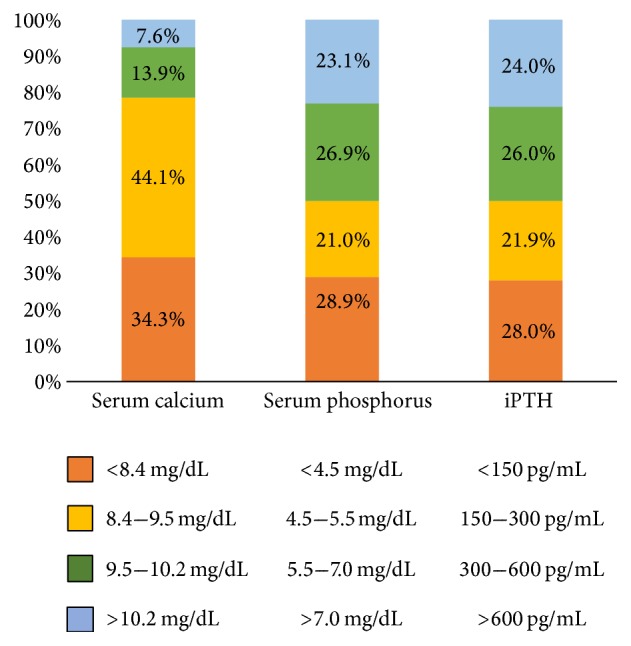
Distribution of serum calcium, phosphorus, and intact parathyroid hormone (iPTH) levels.

**Figure 2:**
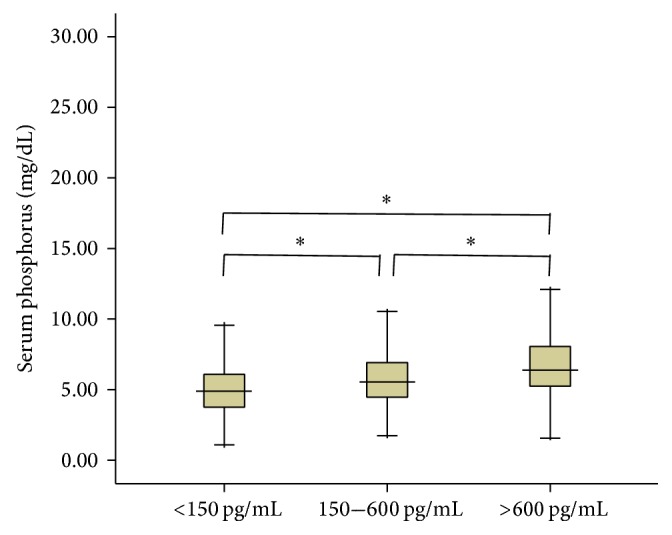
Comparisons of serum phosphorus, according to the different levels of intact parathyroid hormone. Boxes are median and interquartile ranges. Vertical lines represent the 25th to 75th percentile. ^*∗*^*P* < 0.001 using the Mann–Whitney *U* test.

Figure 1 illustrates the distribution of serum Ca, P, and iPTH levels. The percentages of hypocalcemia and hypercalcemia were 34.3% and 21.5%, respectively. The percentage of hyperphosphatemia was 50% and iPTH > 300 pg/mL was also 50%. Elevated iPTH levels showed a significant association with increased hyperphosphatemia (Figure 2). The prevalence of P-binder use was 12.1% and 80% of the P-binders taken were calcium acetate and carbon calcium.

Table 2 shows the general characteristics of the Han and Uyghur groups. Uyghur people were younger than Han people on average (47.02 years versus 55.05 years) and the dialysis vintage was significantly shorter in Uyghur people compared to Han (2.99 years versus 3.44 years). The Ca serum levels, P levels, and iPTH levels were not significantly different. The all-cause mortality rate of the study population was 7.9%, 7.1% in the Han group, and 8.3% in the Uyghur group (data not shown).

## 4. Discussion

This cross-sectional survey is the first report on the status of CKD-MBD markers in HD patients in Xinjiang. There were 3725 patients on HD and the prevalence rate was 162 pmp in Xinjiang, but Han group (234 pmp) was higher than Uyghur group (137 pmp). It may be related that the most of Han group live in urban areas and the most of Uyghur group live in rural areas. Whether it is related to different lifestyle and genetics between Han and Uyghur will be studied. The lower prevalence of HD indicates that the patients of HD will be rapidly increased following economic and medical care improve in Xinjiang during the next 5 years.

In this study, the serum Ca levels in our patients were 8.76 mg/dL, the hypercalcemia rate was 21.5%, and hypocalcemia rate was 34.3%. When compared to DOPPS4 [5, 6], Ca levels in our patients were lower, and hypocalcemia rates were higher. The mean serum P level of our patients was 5.77 mg/dL, higher than DOPPS4 (5.2 mg/dL) [6]. The hyperphosphatemia rate was 50.0% and that was higher than all countries in the DOPPS4 report [5]. The status of CKD-MBD markers in Xinjiang was similar to the Chinese DOPPS report [7, 8]. Interestingly, the overall rate of hypocalcemia and hyperphosphatemia in Xinjiang HD patients was higher than the DOPPS report. We speculated that one of the causes of this might be related to an inadequate number of HD sessions provided to Xinjiang patients. The mean number of HD sessions per week was 2.57 sessions for our patients, which is obviously less than the three sessions per week undertaken in most of the countries included in DOPPS. Another possible cause may have been nephrology teams that are inexperienced in CKD-MBD management given the fairly recent introduction of HD therapy in most areas in Xinjiang. Studies involving longer follow-up periods in Xinjiang are needed to clarify trends in CKD-MBD management in the near future.

In the present study, serum iPTH levels were higher than those reported in the DOPPS [6] and Chinese DOPPS [7]. This result was in accordance with the higher rates of hyperphosphatemia and hypocalcemia seen in Xinjiang HD patients. Based on an analysis of DOPPS, there is an increased risk of death when serum iPTH levels are higher than 600 pg/mL [9]. The K/DIGO clinical guidelines suggest that the optimal iPTH level in laboratory examinations is two to nine times the upper normal limit [4]. In our study, 24% of HD patients had iPTH levels higher than 600 pg/mL. In addition, 47.9% of HD patients met the KDIGO target for iPTH levels. We also noted that only 12% of HD patients were using P-binders in Xinjiang compared to 70% of users in the DOPPS report. Accordingly, a multidiscipline CKD-MBD care program is urgently needed in Xinjiang.

Uyghur people represent 47.8% (11 million) of the total population of Xinjiang; they have a unique genetic background and live with an unusual lifestyle [10]. In the present study, the proportions of glomerulonephritis in Uyghur was higher and diabetic nephropathy was lower than Han; it may be related to the fact that the most part of Uyghur' patients came from rural area (economic status and healthcare levels were lower than those in urban areas). Although Uyghur people demonstrated a higher predialysis blood pressure and lower hemoglobin levels, albumin levels, urea reduction ratios, and *Kt*/*V* values compared to Han people, nevertheless, there were no significant differences in the CKD-MBD markers in the Uyghur and Han groups. This finding indicates that a more aggressive medical strategy is needed to manage Uyghur HD patients according to the guidance for diagnosis and treatment of MBD in CKD by Chinese Society of Nephrology in 2013 [11, 12]. The effect of dietary habits, HD dose, and drug compliance and so forth on unfavorable clinical parameter results needs to be validated by further study on Uyghur people.

Our study had several limitations. First, some information related to CKD-MBD was not available to us, such as detailed data on nutritional intake, particularly dietary phosphorus intake, residual renal function, and accurate amount of P-binders and vitamin D analogs taken by patients. Second, the clinical parameters and CKD-MBD markers were not tested or collected at regular intervals during the study period and a cause-effect relationship cannot be obtained in the present study by single-point observational design. This is a retrospective study which limits the generalization of its findings. Despite these limitations, the present study is the first to investigate the clinical epidemiology of CKD-MBD in the Xinjiang area. Given that it is the largest administrative area in China, clinical investigation of Xinjiang HD patients provides a preliminary picture of CKD-MBD status in Xinjiang. Accordingly, a more effective strategy for medical care in dialysis patients in Xinjiang can be developed based on our results.

## 5. Conclusions

In summary, the present study demonstrated higher serum P and iPTH levels in Xinjiang HD patients than those reported in the DOPPS4 and the Chinese DOPPS. A lower percentage of P-binder use was also noted. A multidiscipline CKD care program needs to be established to improve CKD-MBD target achievement in Xinjiang.

## Figures and Tables

**Table 1:** General patients characteristics (*n* = 3725).

Characteristics	*N* (%)
Age (years)	52.09 ± 15.70
Male	2418 (64.9)
Dialysis vintage	
≤1 year	913 (24.5)
1~3 years	1542 (41.4)
3~5 years	756 (20.3)
5~10 years	447 (12.0)
>10 years	67 (1.8)
Primary kidney diseases	
Primary glomerulopathy	1579 (42.4)
Diabetic nephropathy	861 (23.1)
Hypertensive renal damage	574 (15.4)
Polycystic kidney disease	56 (1.5)
Kidney stone	37 (1.0)
Others	618 (16.6)
HD frequency (times/week)	2.57
Predialysis SBP (mmHg)	148 ± 22
Predialysis DBP (mmHg)	87 ± 14
Body mass index (kg/m^2^)	21.75 ± 5.13
Urea reduction ratio	59.36 ± 17.61
*Kt*/*V*-urea	1.13 ± 0.51
Hemoglobin (g/L)	102.24 ± 22.32
Creatinine (*μ*mol/L)	840.07 ± 329.2
Albumin (g/L)	38.08 ± 6.00
Calcium (mg/dL)	8.76 ± 1.24
Phosphate (mg/dL)	5.77 ± 2.20
iPTH (pg/mL)	468.81 ± 519.5

**Table 2:** General characteristic of Han and Uygur.

Characteristics	Han (*N* = 2352)	Uygur (*N* = 1373)	*X* ^2^/*t*	*P*
Male	1497 (63.6)	921 (67.1)	4.609	0.032
Age (y)	55.05 ± 15.33	47.02 ± 15.01	3.811	<0.001
Dialysis vintage (years)	3.44 ± 2.63	2.99 ± 2.13	27.662	<0.001
HD frequency (times/week)	2.56	2.59	2.203	0.138
Predialysis SBP (mmHg)	146 ± 21	151 ± 22	27.231	<0.001
Predialysis DBP (mmHg)	85 ± 13	90 ± 14	57.208	<0.001
Body mass index (kg/m^2^)	21.70 ± 4.98	21.85 ± 5.38	0.365	0.546
Urea reduction ratio	61.64 ± 15.77	55.13 ± 19.95	54.817	<0.001
*Kt*/*V*-urea	1.18 ± 0.48	1.05 ± 0.56	24.547	<0.001
Hemoglobin (g/L)	104.03 ± 21.55	98.65 ± 23.41	32.466	<0.001
Creatinine (*μ*mol/L)	841.42 ± 312.46	837.26 ± 361.93	0.072	0.789
Albumin (g/L)	38.44 ± 5.97	37.37 ± 6.02	12.569	<0.001
Calcium (mg/dL)	8.92 ± 1.20	8.40 ± 1.16	2.155	0.032
Phosphate (mg/dL)	5.80 ± 0.73	5.67 ± 2.08	0.668	0.504
iPTH (pg/mL)	457.72 ± 521.83	500.45 ± 512.41	1.560	0.120
